# Chagasic Thymic Atrophy Does Not Affect Negative Selection but Results in the Export of Activated CD4^+^CD8^+^ T Cells in Severe Forms of Human Disease

**DOI:** 10.1371/journal.pntd.0001268

**Published:** 2011-08-16

**Authors:** Alexandre Morrot, Eugênia Terra-Granado, Ana Rosa Pérez, Suse Dayse Silva-Barbosa, Novica M. Milićević, Désio Aurélio Farias-de-Oliveira, Luiz Ricardo Berbert, Juliana De Meis, Christina Maeda Takiya, Juan Beloscar, Xiaoping Wang, Vivian Kont, Pärt Peterson, Oscar Bottasso, Wilson Savino

**Affiliations:** 1 Department of Immunology, Microbiology Institute, Federal University of Rio de Janeiro, Rio de Janeiro, Brazil; 2 Laboratory on Thymus Research, Oswaldo Cruz Institute, Oswaldo Cruz Foundation, Rio de Janeiro, Brazil; 3 Institute of Immunology, School of Medical Sciences, National University of Rosario, Rosario, Argentina; 4 Department of Clinical Research, National Cancer Institute, Rio de Janeiro, Brazil; 5 Institute of Histology and Embryology, Faculty of Medicine, University of Beograd, Beograd, Serbia; 6 Biomedical Science Institute, Cell Pathology Laboratory, Federal University of Rio de Janeiro, Rio de Janeiro, Brazil; 7 Molecular Pathology, Institute of General and Molecular Pathology, University of Tartu, Tartu, Estonia; National Institutes of Health, United States of America

## Abstract

Extrathymic CD4^+^CD8^+^ double-positive (DP) T cells are increased in some pathophysiological conditions, including infectious diseases. In the murine model of Chagas disease, it has been shown that the protozoan parasite *Trypanosoma cruzi* is able to target the thymus and induce alterations of the thymic microenvironment and the lymphoid compartment. In the acute phase, this results in a severe atrophy of the organ and early release of DP cells into the periphery. To date, the effect of the changes promoted by the parasite infection on thymic central tolerance has remained elusive. Herein we show that the intrathymic key elements that are necessary to promote the negative selection of thymocytes undergoing maturation during the thymopoiesis remains functional during the acute chagasic thymic atrophy. Intrathymic expression of the autoimmune regulator factor (Aire) and tissue-restricted antigen (TRA) genes is normal. In addition, the expression of the proapoptotic Bim protein in thymocytes was not changed, revealing that the parasite infection-induced thymus atrophy has no effect on these marker genes necessary to promote clonal deletion of T cells. In a chicken egg ovalbumin (OVA)-specific T-cell receptor (TCR) transgenic system, the administration of OVA peptide into infected mice with thymic atrophy promoted OVA-specific thymocyte apoptosis, further indicating normal negative selection process during the infection. Yet, although the intrathymic checkpoints necessary for thymic negative selection are present in the acute phase of Chagas disease, we found that the DP cells released into the periphery acquire an activated phenotype similar to what is described for activated effector or memory single-positive T cells. Most interestingly, we also demonstrate that increased percentages of peripheral blood subset of DP cells exhibiting an activated HLA-DR^+^ phenotype are associated with severe cardiac forms of human chronic Chagas disease. These cells may contribute to the immunopathological events seen in the Chagas disease.

## Author Summary

The thymus is a primary lymphoid organ that plays an important role on the development of the immune system and maturation of the T cell repertoire. During the normal life span, this organ undergoes involution during the aging and also in the presence of a wide variety of infectious diseases. It has been shown that the protozoan parasite *Trypanosoma cruzi* is able to target the thymus and induce alterations of the thymic microenvironment. In the acute phase, this results in a severe atrophy of the organ and early release of immature double-positive (DP) T cells into the periphery. The effect of the changes promoted by the parasite infection on thymic central tolerance has remained not clear. The present study shows that the intrathymic key elements that promote the negative selection of thymocytes during the thymopoiesis remains functional in the acute chagasic thymic atrophy. However, we found that the DP cells released into the periphery acquire an activated phenotype and its high frequency in the peripheral blood are associated with severe cardiac forms of human chronic Chagas disease.

## Introduction

Chagas disease is caused by the flagellate protozoan *Trypanosoma cruzi* (*T. cruzi*) and represents an important public health burden in the Americas. After infection, the initial acute phase of the disease progresses to an asymptomatic indeterminate period with virtually undetectable parasitemia. The patients have a strong humoral and cellular anti-*T. cruzi* responses, resulting in non-sterile control of the parasite. Up to several years after the initial infection, approximately 20 to 30% of all infected individuals develop a chronic inflammatory disease primarily affecting the heart [1]. The pathogenesis of Chagas disease is controversial and distinct hypotheses have been considered, including autoimmune manifestations and parasite-driven tissue damage [2]. In any case, it is accepted that the events occurring during the acute phase of *T. cruzi* infection are determinant for the pathological features to be settled later, during the chronic phase of the disease [3].

In experimental models of Chagas disease, several alterations in lymphoid organs were observed, including the thymus where the parasite has been detected [1]. Previous studies have revealed a severe thymic atrophy in acutely infected animals mainly due to apoptotic depletion of CD4^+^CD8^+^ double-positive (DP) thymocytes in the cortical area of the thymic lobules [4], [5]. Thymocyte depletion parallels *T. cruzi*-induced alterations of the thymic microenvironment, comprising phenotypic and functional changes in the thymic epithelial cell (TEC) network, with enhancement in the deposition of cell migration-related molecules such the extracellular matrix (ECM) proteins, fibronectin and laminin, as well as the chemokines CXCL12 and CCL21 [6]–[8]. These changes are likely related to the abnormal release of DP cells from the thymus into the periphery, resulting in more than 15 fold increase in DP cell numbers in subcutaneous lymph nodes. In this vein, it is noteworthy that DP cells seen in peripheral lymphoid organs express high densities of ECM and chemokine receptors [9]. Among these abnormally released DP cells in the periphery, we found lymphocytes expressing potentially autoreactive TCRs, which are normally deleted in the thymus of uninfected mice. This suggests that during the infection, immature T lymphocytes escape from the thymic central tolerance process and migrate to the lymph nodes where they eventually differentiate into mature CD4^+^ or CD8^+^ cells [9], [10].

During lymphocyte development, TCR gene assembly occurs by combinatorial linking of gene segments. The nature of this process gives rise to lymphocytes that can recognize self-antigens and have the potential to induce autoimmunity [11]. In the thymus, potentially autoreactive lymphocytes are induced to undergo apoptotic cell death during the negative selection process, which involves the elimination of potentially autoreactive T cell clones bearing high affinity TCR that recognize autoantigens presented by TEC. Intrathymic expression of tissue-restricted antigens (TRAs), activated by the autoimmune regulator (Aire) protein in TECs, has an important role in the induction of negative selection of thymocytes [12]. The Aire protein has several domains that are characteristic to transcriptional activators and has been reported to interact with a common transcriptional regulator, the CREB binding protein (CBP) [13], [14]. In turn, the apoptosis mechanism in thymocytes is dependent on the activation of the pro-apoptotic Bcl-2 family member Bim. The TCR ligation upregulates the BH3-only protein Bim expression and promotes interaction of Bim with Bcl-X_L_, inhibiting its survival function [15]–[17]. Loss of either Aire or Bim expression results in altered thymic deletion of autoreactive lymphocytes and has been implicated in autoimmune manifestations [18]–[21].

Herein we attempted to clarify whether the changes in the thymic microenvironment, as a result of parasite infection, play a role on the modulation of the negative selection of thymocytes, a key element for the control of autoimmunity. To this end, we analyzed the intrathymic expression of Aire and TRAs as well as the expression of pro-apoptotic Bim protein during the course of the thymic *T. cruzi* infection. Using the ovalbumin specific DO11.10 TCR transgenic system, we further investigated whether the transgenic thymocytes are hypersensitive to stimulation with the specific OVA peptide in mice undergoing *T. cruzi* promoted-thymic atrophy. In addition, we analyzed the activation profile of the DP cell subset that is prematurely released to the periphery during the course of the infection. Our study reveals that regardless of thymic changes promoted by the *T. cruzi* infection, the negative selection is still functional during the acute infection of the parasite. However, we show that in contrast to the physiological condition, the DP cells released into the periphery during the course of the infection acquire an activated phenotype similar to what is described for activated single-positive T cells. Furthermore, we show that the presence activated DP cells in the periphery is correlated with the development of severe clinical form in chronic human Chagas disease.

## Materials and Methods

### Ethics statement

The study was approved by the Research Ethics Committee of National University of Rosario, (protocol UNR-CD 2854/2008) and Fiocruz (protocol CEUA-LW8/10). Protocols for animal and human studies were approved by the Institutional Ethical Committees in accordance with international guidelines. All animal experimentation was performed in accordance with the terms of the Brazilian guidelines for the animal welfare regulations. All individuals provided written informed consent.

### Study population

Healthy volunteers and *T. cruzi* chronic chagasic patients were recruited from Chagas Unit, Hospital Provincial del Centenario de Rosario, UNR, Argentina. Subjects using any medication thought to affect immune functions were excluded from the study. The diagnosis was based on standard serological test, including indirect immunofluorescence and haemaglutination assay. All chronic infected patients and noninfected individuals participated in serological tests to confirm the diagnosis for *T. cruzi* infection, with ages ranging from 30 to 64 years. The seropositive cases included fifteen cardiac chronic-infected patients presenting dilated cardiomyopathy diagnosed based in a detailed clinical examination, including electrocardiography (ECG) and chest X-ray. Additionally, we included fifteen chagasic patients without any cardiac alterations detected, being diagnosed as in the indeterminate form of the disease. Fifteen sex and age matched-controls were also included.

### Animals and infection

Male BALB/c mice, aged 4–8 weeks, were obtained from the Oswaldo Cruz Foundation animal facilities. The OVA-TCR transgenic mice (BALB/c background, clone D011.10, that recognize the 323–339 peptide fragment of OVA in the context of I-A^d^), were bred under standard conditions. Acute *T. cruzi* infection was performed by inoculating the mice intraperitoneally with 10^2^ blood-derived trypomastigote forms of the *Tulahuén* strain. After 2 weeks of acute infection (at the peak of parasitemia), animals were bled and sacrificed, and the organs to be studied were removed. Blood parasites were counted using Neubauer's chambers.

### Tissue preparation, silver impregnation and histochemistry

Thymuses from normal and infected animals (after 2 weeks of acute infection) were fixed in neutral buffered formaldehyde or Bouin's solution and processed for paraffin wax sectioning. The 3–5 µm thick paraffin sections were stained with hematoxylin-eosin, impregnated with ammoniacal silver according to Weil–Davenport method or stained with Periodic acid-Schiff for demonstration of metallophilic macrophages, as described [45].

Immunoperoxidase assays were performed using standard techniques on 4% formaldehyde-fixed frozen sections or chamber slides. Endogenous peroxidase was blocked with 3% H_2_O_2_. Non-specific protein binding was blocked by incubation with 5% normal swine serum. Sections were sequentially incubated with rabbit anti-mouse Aire polyclonal antibody for 60 min at 37°C, horseradish peroxidase (HRP) labeled swine anti-rabbit polyclonal antibody for 60 min at 37°C, with intervening phosphate buffer solution (PBS) washes. After a complete wash in PBS, the slides were developed in 0.05% freshly prepared 3,3′-diaminobenzedine solution with 0.03% hydrogen peroxide for 5 min, and then counterstained with hematoxylin, dehydrated, air-dried, and permanently mounted. For frozen sections, the optimum dilution of each antibody was first determined by serial dilutions on normal mouse thymus sections. Normal swine serum was used to substitute for the primary antibody to serve as a negative control. The stained cells were observed under a Bio-Rad MRC 1024ES regular microscope equipped with a Zeiss Diaphot inverted microscope (Jena, Germany). Rabbit anti-mouse Aire polyclonal antibody and HRP labeled swine anti-rabbit polyclonal antibody were purchased from Santa Cruz Biotechnology Inc. (California, USA).

### Immunoprecipitation and western blotting analysis

Thymocytes were lysed at a concentration of 3×10^8^ cells/ml in 0.2% Nonidet P-40 lysis buffer. Lysates were incubated on ice for 15 min, centrifuged at 16,000 x *g* for 5 min at 4°C, and pre-cleared with protein A/G beads. Bim was immunoprecipitated from lysates with 3 µg of monoclonal rat anti-Bim clone 3C5 Ab, gently provided by Professor Andreas Strasser (The Walter and Eliza Hall Institute of Medical Research, Australia). Control lysates were incubated with 3 µg of normal rabbit IgG (Santa Cruz Biotechnology). The lysate mixtures with antibodies were gently rocked at 4°C for 1 h. Protein A/G beads (50 µl of a 50% (v/v) slurry) were added to each sample and rocked at 4°C for an additional hour. After incubation, the beads were washed with lysis buffer three times, then washed with PBS and transferred to fresh tubes. The bound proteins were eluted with nonreducing 4x SDS sample buffer by boiling for 5 min and subjected to Western blot analysis.

### Real-time PCR

RNA was isolated using TRIzol (Invitrogen, Life Technologies) and reverse-transcribed to cDNA using the SuperScript TM III Reverse Transcriptase (Invitrogen, Life Technologies). Real-time PCR was performed with the ABI Prism 7900HT Fast Real-Time PCR System instrument (Applied Biosystems) using the qPCR SYBR Green Core Kit (Eurogentec) according to the manufacturer's instructions, except that 2 mM MgCl2 concentration was used. The amplification program included an initial denaturation step at 95°C for 10 min, followed by denaturation at 95°C for 15 s, and annealing and extension at 60°C for 1 min, for 45 cycles. SYBR Green fluorescence was measured after each extension step, and the specificity of amplification was evaluated by melting curve analysis. Primers used to amplify specific gene products from murine cDNA were K5 sense, 5′-agtcaacatctccgtcgtcac-3′; K5 antisense, 5′-gggactgcctaaaagaagcag-3′; Aire 11/12, 5′-ccccgccggggaccaatctc-3′; Aire 12/13, 5′-agtcgtcccctaccttggcaagc-3′; Ins2 sense, 5′-gacccacaagtggcacaac-3′; Ins2 antisense, 5′-tctacaatgccacgcttctg-3′, Mup1 sense, 5′-tctgtgacgtatgatggattcaa-3′; Mup1 antisense, 5′-tctggttctcggccatagag-3′; Spt1 sense, 5′-aacttctggaactgctgattctg-3′; Spt1 antisense, 5′-gaggcctcattagcagtgttg-3′; IFN-γ sense, gaaagcctzgaaagtctgaataact; IFN-γ antisense, atcagcagcgactccttttccgct; GAPDH sense, gccgcctggagaaacctcccaagt; GAPDH antisense, tattcaagagagtagggagggctc. The relative gene expression levels were calculated using the comparative *C*t method (according to Applied Biosystems), where *C*t represents the threshold cycle. Every sample was run in three parallel reactions.

### Isolation of peripheral blood cells (PBMC), antibodies and flow cytometry

Heparinized whole blood was collected and diluted 1/1 with PBS before separated by density centrifugation on Ficoll-Hypaque (Sigma) for 30 min at 2000 rpm. All antibodies were purchased from BD Biosciences (CA, USA). For human T cell phenotyping, individual samples contained 1.10^6^ living cells were pre-blocked with human AB serum for 15 min and stained at 4°C for 30 min simultaneously with four colors using the following antibodies for FACS analysis: APC-labeled anti-CD3, APCCy7-labeled anti-CD4, PECy7-labeled anti-CD8 and FICT-labeled anti HLA-DR antibodies (BD/PharMingen). For murine T cell phenotyping experiments, thymuses and subcutaneous lymph nodes (pool of inguinal, axilary and brachial lymph nodes) were removed from infected and control animals. The organs were minced, washed and resuspended in PBS-FCS 5% for subsequent evaluation of cellularity, which was followed by triple or quadruple immunofluorescence staining, as previously described [8]. Cells were then fixed and analyzed by flow cytometry in a FACSCalibur flow cytometer. Analyses were done after recording 25,000–50,000 events for each sample, using a CELLQuest software (Becton Dickinson). Lymphocytes were gated based on forward and side scatter parameters, so as to avoid larger leukocytes such as macrophages and granulocytes.

### Isolation of DP cells from lymphoid tissues and quantification of mouse IFN-γ mRNA transcripts

CD4^+^CD8^+^ T cells were obtained from lymphoid tissues of normal and infected animals in the peak of parasitemia (2 weeks post-infection). The total number of CD4^+^CD8^+^ T cells was determined by FACS analysis. The purification of the DP subset was obtained by FACS cell sorting using anti CD3-FITC, anti CD4-PERCP, and anti CD8-PE. After FACS cell sorting, the total RNA from DP subset was extracted using standard Trizol extraction. mRNA transcripts for mouse IFN-γ were amplified using specific primers in quantitative RT-PCR as described [46].

### CFSE-based cytotoxic assay

The mouse mastocytoma cell line, P-815, was labeled with CFSE based on the manufacturer's instructions (Molecular Probes) and used as target cells. The labeled cells were adjusted to 5×10^4^ cells/mL, and 100 µL/well was plated in 96-well microtiter plates. CD4^+^CD8^+^ T cells obtained from lymphoid tissues of infected animals or normal single-positive CD4^+^ or CD8^+^ T cells as controls were added at different effector-target ratios. Plates were incubated in a humidified atmosphere of 5% CO_2_ and 37°C in the presence of anti-CD3 mAb at a concentration of 20 µg/ml for TCR triggered-redirected cytotoxicity activity. After 6 hours, the wells were harvested to allow quantitative analysis of the cell populations by FACS analysis. To stain for dead cells, propidium iodide (1 µg/mL) was added, and samples were mixed properly and directly analyzed by flow cytometry. The absolute number of surviving cells was determined at each time point by calculation of the acquisition of CFSE-lived cells at a fixed period of time. The absolute number of cells at the moment of T- cell addition (t = 0) is the number of target cells added to the wells (5,000). The percentage of survival was calculated as follows: % survival  =  [absolute no. viable CFSE^+^ target cells (t = x)]/[absolute no. viable CFSE^+^ target cells (t = 0)] x 100.

### Statistical analysis

Results were expressed as mean ± standard error, unless otherwise indicated. Parametric and non parametric tests were used, depending on the characteristics of variables (normal distribution or not, etc). Comparisons among groups were made by parametric analysis of variance (k groups >2) followed by Student's t-test (k groups  = 2) or non parametric Kruskall-Wallis test (k groups >2) followed by the U Mann-Whitney test (k groups  = 2). Differences between control *versus* infected groups were considered statistically significant when *P*≤0.05.

## Results

### Thymic atrophy following acute *Trypanosoma cruzi* infection

**Figure 1:**
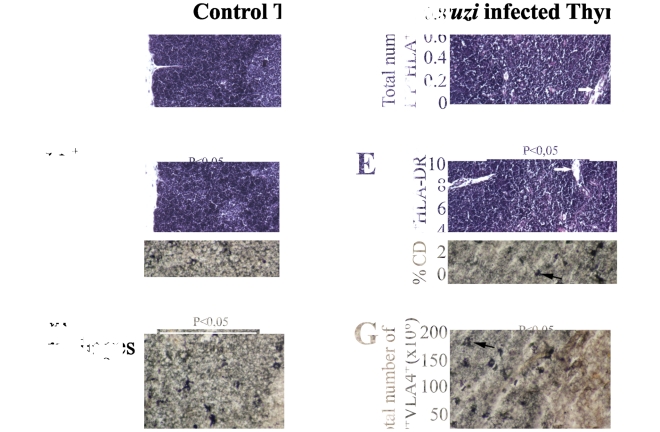
Thymic atrophy in BALB/c acutely infected with *T. cruzi*. The histological profiles show the **(upper left)** normal thymic architecture (C, cortex; M, medulla) and **(upper right)** marked cortical and medullary atrophy in the thymus of an acutely-infected mouse. **(lower left)** The numerous metallophilic macrophages are present in the cortico-medullary zone (arrows) of normal thymus. However, following *T. cruzi* acute infection **(lower right)** the number and distribution of metallophilic macrophages are changed with the cells dispersed throughout not only the cortico-medullar area but also the cortical region. Infected mice were evaluated herein at day 15 post-infection. These data are representative of two independent experiments using four mice per group.

The acute infection experimental model of *T. cruzi* induces a severe thymic atrophy that is evident at 10 days post-infection after the intraperitoneal injection of BALB/c mice with 10^2^ *Tulahuén* trypomastigote parasites. In this model, the parasitemia progresses with a peak at day 14, and 20 days after infection, when the animals start to succumb. The thymuses of normal BALB/c mice show a well-developed cortex and medulla with a clear cortico-medullary boundary, delicate capsular and septal tissue and medullary blood vessels of usual appearance (Fig. 1, upper left panel). In the acute model of Chagas disease, the thymuses exhibit progressive atrophy of the cortex and medulla with a blurred cortico-medullary boundary region. In addition, rough and broadened capsular and septal connective tissue, and more numerous and dilated medullary blood vessels can be observed during the course of infection (Fig. 1, upper right panel).

### Metallophilic macrophages are dispersed in infected thymus

As the macrophages represent a parasite reservoir in the thymus [22], we evaluated whether the thymic changes observed during the infection could compromise the viability of macrophages and their localization in the thymic microenvironment. The metallophilic macrophages correspond to a special type subpopulation of mature resident macrophages present with prominent dendrites in the thymus. They are distinguished by a high non specific esterase activity and can be stained with silver impregnation [23], and have an important role in the process of thymocyte maturation, being able to present antigens by both MHC class I and II molecules [23], [24]. Accordingly, they are particularly active in the process of negative selection, which is in keeping with their strategic localization within thymic parenchyma, forming a distinct row of cells at the cortico-medullary junction of the thymic lobules [23].

Interestingly, in contrast to normal thymus (Fig. 1, lower left panel), we found that in the infected thymus, numerous metallophilic macrophages are dispersed not only in the cortico-medullary area but also throughout the cortical compartment, appearing as large cells with abundant cytoplasm (Fig. 1, lower right panel). Nevertheless, in infected thymuses we noted the presence of these cells at the cortico-medullary junction where the clonal deletion is believed to occur. As the metallophilic macrophages are mostly participating in the uptake of dying thymocytes, but not in induction of apoptosis that is mediated by DC or medullary epithelial cells, it suggests that their altered location in cortical areas is not necessarily affecting the thymic clonal deletion.

### Expression of Aire and tissue-restricted antigens in the *Trypanosoma cruzi* infected thymus

Previous studies have demonstrated that TRAs are expressed in the thymus and this expression is needed for the deletion of self-reactive T cells. An important molecule in regulation of TRA expression by medullary TEC (mTEC) is Aire [12]. Several studies have shown the Aire protein to be predominantly expressed in TEC and suggest it has a role in regulation of immune tolerance by modulating the TRA expression in the thymus [25]. Therefore, we explored whether the acute atrophy of the thymus induced by the *T. cruzi* infection could modulate the expression of TRAs and Aire in the thymus. For this purpose, we collected the thymuses at day 15 post-infection when the atrophy is severe and compared the expression of Aire and TRAs in infected and normal thymus by real-time PCR.

To study the expression of self-antigens we chose three TRAs (Ins1, Mup1 and Spt1) that are downregulated in Aire deficient mice and follow the same expression pattern as the Aire gene. All three genes have highly selective, tissue-specific expression. For example, Ins1 encodes for insulin expressed by pancreatic beta cells, Spt1 is expressed in salivary and lacrimal glands, and Mup1 is expressed in the liver and also in salivary, lacrimal and mammary glands [25]. The results were normalized to the expression level of keratin 5 (k5) mRNA, which is specifically expressed in medullary thymic epithelial cells [26].

**Figure 2:**
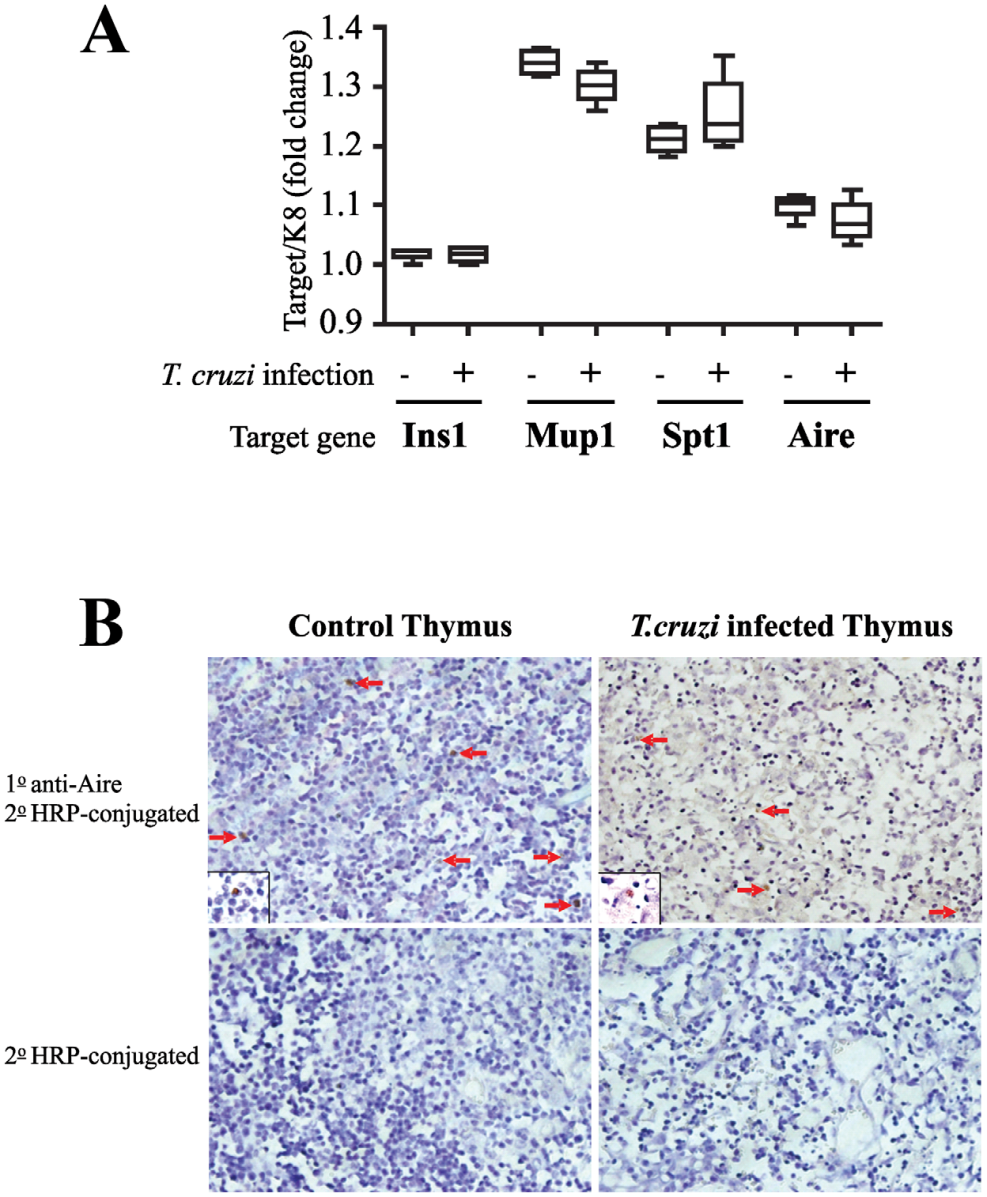
Intrathymic tissue-restricted antigen expression levels in atrophic thymus during *T cruzi* infection. **(a)** Thymuses were collected from normal or *T. cruzi* infected BALB/c (day 15 post-infection) and expression levels of Aire and TRA genes were analyzed by real-time PCR. The expression of Aire as well as TRA genes was comparable in infected and uninfected thymus. Data are mean ± standard error. of triplicate measurements in one of two representative experiments using five mice per group. **(b)** Thymuses were stained with anti-Aire antibody and analyzed by immunofluorescence. The histological profiles show the presence of Aire-positive cells (arrows) in **(upper left)** normal thymus and **(upper right)** atrophic thymus at day 15 post-infection. The respective staining controls without the primary antibodies are represented in **(lower left)** and **(lower right)**. Inserts represent higher magnifications of Aire-positive cells (brown).

The real-time PCR analysis showed similar levels of Aire expression in both normal and infected thymus (Fig. 2a). To more accurately assess the protein expression of Aire within the thymus, in situ immunohistochemical staining with anti-Aire polyclonal antibody was performed in thymus sections. Despite the atrophy observed during the acute infection, we did observe Aire^+^ cells in thymuses at day 15 post-infection as compared to physiological condition (Fig. 2b). This profile was followed by the expression of TRA genes, showing that the expression of ectopic genes was unaffected by *T. cruzi* infection as compared to the control mice. Collectively these data show that both Aire and TRAs are normally expressed in the thymus of *T. cruzi* infected mice.

### Intrathymic proapoptotic Bim expression levels in *Trypanosoma cruzi* acute infection

**Figure 3:**
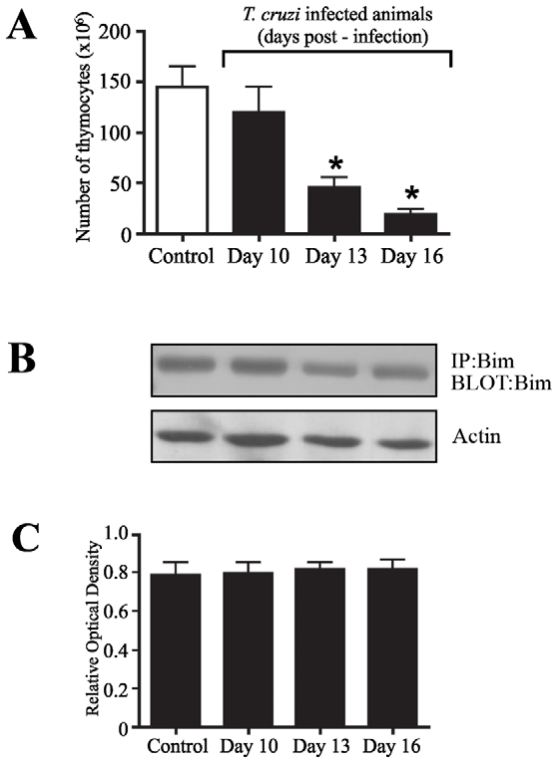
Intrathymic expression of Bim in the course of acute *T. cruzi* infection. **(a)** Thymuses were collected from normal or *T cruzi* infected BALB/c mice (day 15 post-infection). All numbers are given in 10^6^, and each value represents the mean of total thymocyte numbers in 4-8 mice per group. **(b)** From individual groups of the same experiments, DP cells isolated by cell sorting from thymus of infected mice at the indicated time-points or normal mice as controls were lysed and proteins were solubilized by detergent lysis and immunoprecipitated with anti-Bim antibody for Western blotting analysis probing with the same antibody. The upper blot shows kinetics of the Bim expression from day 10 to 16 post-infection, during the thymic atrophy, whereas the lower panel reveals a blot was probed with actin, applied as a loading control. **(c)** Optical densitometry of the western blots using NIH Image software, where Bim expression was normalized with the actin expression. The data are representative of three independent experiments. *Differences between control *versus* infected mice are significant (*P*<0.05). These data are representative of two independent experiments using five mice per group.

In order to evaluate the checkpoints of the intrathymic thymocyte negative selection, we next assessed the proapoptotic Bim expression levels in DP thymocytes during *T. cruzi*-induced thymus atrophy. To analyze Bim expression levels, thymuses were collected from normal and infected mice at the indicated time points, and the viable cells were counted by trypan blue exclusion (Fig. 3a), and the FACS sorted DP cells were analyzed for Bim expression by Western blotting with anti-Bim antibodies. The Bim protein expression was readily detectable in thymocytes obtained from uninfected control mice as seen in Figure 3b. From day 10 to 16 post-infection, we found that the expression levels of Bim in infected mice along the course of thymus atrophy were similar to control animals (Fig. 3b-c), thus indicating that there is no major change in expression level of proapoptotic Bim protein.

### Functional analysis of negative selection in *Trypanosoma cruzi* infected thymus

As the expression of Aire/TRA genes and the pro-apoptotic Bim protein were not changed in the thymus undergoing atrophy upon *T. cruzi* infection, we next wanted to confirm that the negative selection from infected thymus is functional. To address this question, we took advantage of the OVA-transgenic system in which the administration of the cognate antigen promotes the TCR-induced killing of semi-mature thymocytes *in vivo* [27]–[29]. We found that injection of OT II TCR transgenic non-infected mice with the ovalbumin 323–339 cognate peptide (three times every 24 h) significantly decreased the number of DP thymocytes at 3 days after the first injection, which is consistent with the results described earlier [28], [29]. Deletion of immature CD4^+^CD8^+^ DP cells was assessed by counting total thymocyte numbers and by determining the proportions of cell subsets by flow cytometry staining with antibodies to CD4 and CD8 cell-surface markers.

**Figure 4:**
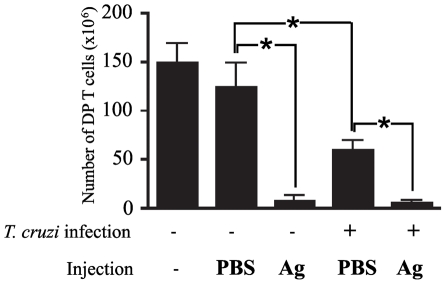
CD4^+^CD8^+^ T cell numbers in thymus of *T. cruzi* infected OVA TCR transgenic mice. OVA-TCR transgenic mice noninfected or infected with *T. cruzi* (9 days post-infection) were injected *i.v.* with 0.5 ml of 450 mM solution of OVAp323–339 daily for 3 days and thymocytes were isolated on the fourth day for quantification of double-positive CD4^+^CD8^+^ T cells by flow cytometry. For control, mice were injected with 0.5 ml of PBS instead of the cognate antigen as indicated in the histogram. All numbers are given in 10^6^. Each value represents the mean of total DP cell numbers in 4-8 mice per group. Data are expressed as mean ± standard error of triplicate measurements of one out of two representative experiments. *Differences between groups are significant (*P*<0.05). These data are representative of two independent experiments using five mice per group.

We then evaluated whether the depletion of DP cells by co-administration of the cognate antigen is affected in the presence of thymus atrophy during *T. cruzi* infection. Similarly to non-infected BALB/c OT II TCR transgenic mice, we observed a drastic reduction of the intrathymic DP cell population in *T. cruzi* infected transgenic mice at day 12 post-infection (Fig. 4). Next, we investigated whether there is a significant change in numbers of DP cells caused by the thymic negative selection of thymocytes undergoing differentiation upon infection. As shown in Figure 3, the deletion of DP cells from infected mice was increased to the levels of control mice upon administration of OVA 323–339 peptides. These results suggest that the changes promoted in the thymus by acute infection of *T. cruzi* do not affect negative selection of DP cells undergoing antigen-driven thymic differentiation.

### CD4^+^CD8^+^ cells acquire an activated/effector phenotype in *Trypanosoma cruzi* infected mice and in chronic chagasic patients

**Figure 5:**
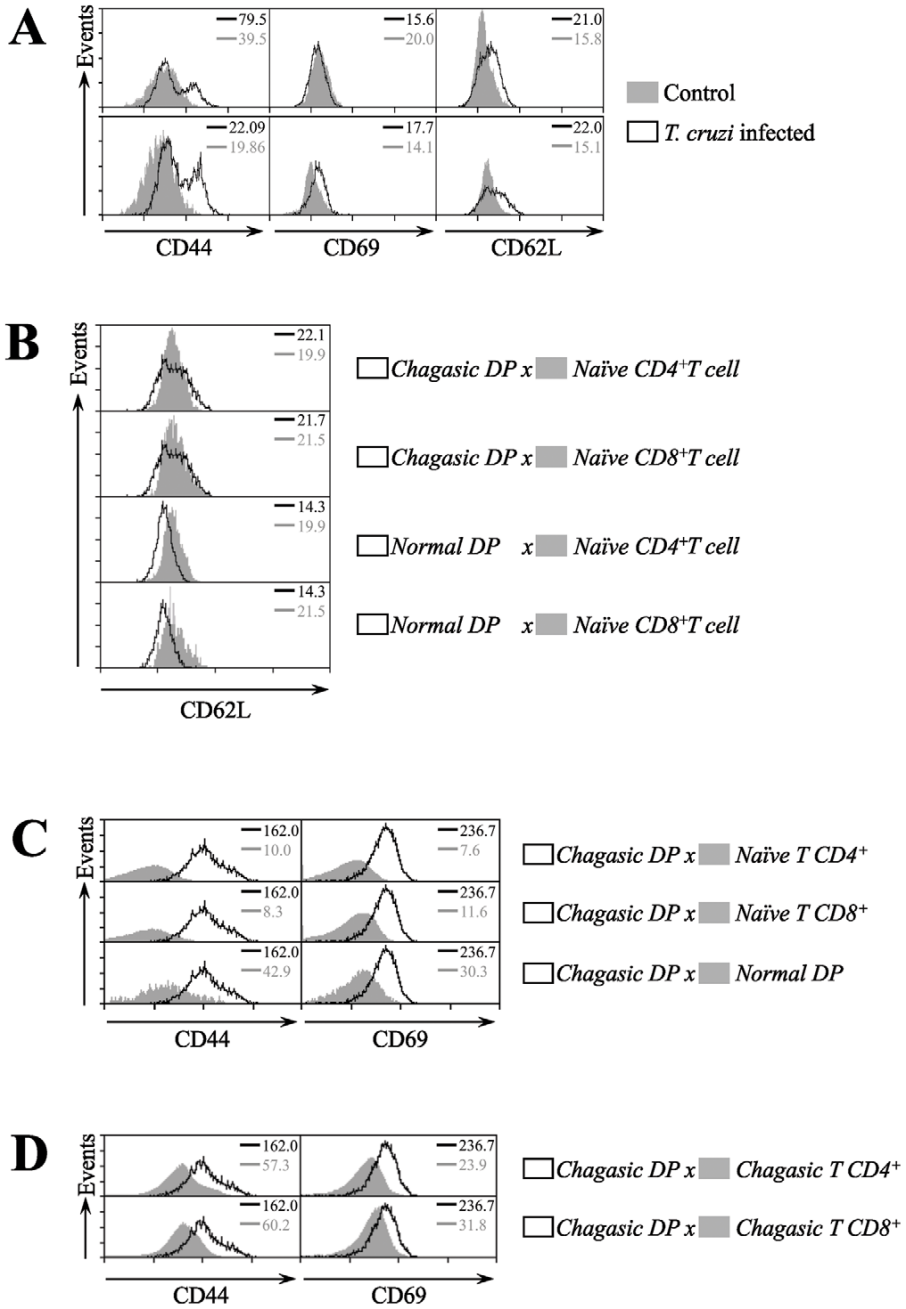
Activation markers in lymphocytes from thymus and subcutaneous lymph nodes of *T. cruzi* infected mice. Mice were infected with *T. cruzi* and 15 days later lymphocytes were isolated from thymus and subcutaneous lymph nodes. **(a)** Thymocytes were stained with CD4-PE, CD8-APC and marker-specific fluorescein isothiocyanate-labeled antibodies prior to flow cytometry analysis. The histograms represent the expression of the CD44, CD69 and CD62L markers in total thymocytes (upper panel) and CD4^+^CD8^+^ T cells (lower panel) from chagasic and normal mice. **(b)** Comparison of the CD62L expression level from normal or chagasic CD4^+^CD8^+^ T cells *versus* normal single-positive CD4^+^ and CD8^+^ T cells undergoing intrathymic maturation. Panel **(c)** depicts representative histograms of CD4^+^CD8^+^ from *T. cruzi* infected subcutaneous lymph nodes (solid lines), compared with naïve CD4^+^ and CD8^+^ T cells obtained from non-infected mice. **(d)** Representative histograms of CD44 and CD69 expression in CD4^+^CD8^+^ T cells, activated single-positive CD4^+^ and CD8^+^ T cells from subcutaneous lymph nodes obtained from *T. cruzi* infected mice. Animals were sacrificed on day 15 post-infection. The data are representative of three independent experiments. The values on the upper right corner indicate the mean fluorescent intensity of the expression of the markers from each histogram. Differences between chagasic DP and activated T cells *versus* naïve T cells are significant (*P*<0.05). These data are representative of three independent experiments using five mice per group.

Since all our findings suggested that negative selection in *T. cruzi* infected thymus was functionally normal, we next focused on the phenotypic characterization of the DP cells that are abnormally released from the thymus upon *T. cruzi* infection. In this respect, we determined whether these cells exhibit an activated profile similar to effector and memory single positive T cells. Using multiparameter FACS analysis we assessed the expression of the major cell surface markers that are known to undergo changes after *in vivo* activation of T cells. In parallel, we phenotyped thymocytes from *T. cruzi* infected or normal mice. We found that DP cells from *T. cruzi* infected thymuses, but not from controls, express high levels of CD44 and CD69. These levels were consistent with those previously described for activated CD4^+^ and CD8^+^ T cells (Fig. 5a).

Moreover, analysis of thymocytes for the expression of L-selectin (CD62L), an up-regulated marker of recent thymic emigrant and mature T cell subsets, indicated that DP cells obtained from acutely chagasic animals express high levels of this molecule, as compared to corresponding controls (Figs. 5a–b). In fact, our data show that CD62L expression levels of intrathymic DP cells obtained from chagasic animals (but not from normal mice) are comparable to the normal single-positive CD4^+^ and CD8^+^ T cells that mature and are exported from the thymus (Fig. 5b). When comparing the activation profile of DP cells obtained from peripheral lymphoid tissues at day 15 after *T. cruzi* infection, the surface markers CD44 and CD69 were highly expressed as compared to naïve CD4^+^ and CD8^+^ T cells (Fig. 5c). We also determined that the DP cells released from thymus during the organ atrophy showed a characteristic profile of CD44 and CD69 expression similar to single-positive T cells differentiated upon infection with *T. cruzi* (Fig. 5d).

**Figure 6:**
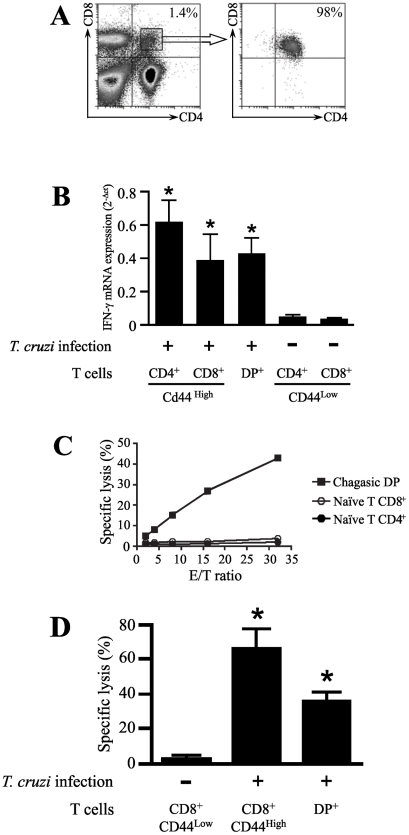
mRNA levels for IFN-γ and cytotoxic activity of peripheral DP T cells during *T. cruzi* infection. **(a)** CD4^+^CD8^+^ T cells from acutely-infected mice were purified from subcutaneous lymph nodes 15d after infection, by cell sorting using flow cytometry. **(b)** To assess IFN-γ transcriptional levels, total mRNA from highly purified fresh DP, CD4^+^ and CD8^+^ T cells (as indicated) were processed for RT-PCR. The results represent the amount of IFN-γ transcript, depicted in relation to the housekeeping gene GAPDH as 2^-ΔCt^ and presented as mean values ± SE. **(c)** Peripheral DP T cells isolated from *T. cruzi* infected mice showed anti-CD3 antibody-redirected cytotoxic activity. P815 target cells were labeled with CFSE, and the cytotoxic activity was assessed by counting the number of target cells by FACS after 6 hours at 37°C. A cytofluorometric-based cytotoxic assay was performed, P-815 cells were labeled with CFSE and then reacted with anti-CD3 or no antibodies, as control for determination of spontaneous activity. The P-815 cells (5×10^3^ cells/well) were added in 96-well round bottom plates. DP, naïve single-positive CD4^+^ or CD8^+^ T cells were added to target cells at the E:T ratios of 2∶1, 4∶1, 8∶1, 16∶1 and 32∶1. **(d)** Comparison of the cytotoxic activity from DP cells and activated CD8^+^ T cells purified from subcutaneous lymph nodes 15d after infection *versus* naïve CD8^+^ T cells obtained from non-infected animals as control. Cytotoxic activity was calculated as described in methods. All experiments were performed in triplicate and data shown are the means. Representatives are shown from two independent experiments. *Differences between DP and activated T cells vs. naïve T cells are significant (*P*<0.05). These data are representative of two independent experiments using five mice per group.

To further compare the differentiation of DP cells with activated cells, we determined the changes in level of IFN-γ transcripts after *T. cruzi* infection. Total RNA was isolated from DP cells sorted by FACS from peripheral lymph nodes 15 days after infection (Fig. 6a). The positive controls were obtained from activated CD4^+^ and CD8^+^ T cells purified from peripheral lymphoid tissues at day 15 after *T. cruzi* infection by FACS cell sorting based on the expression of high levels of the CD44 activation marker. As negative control, we purified naive CD4^+^ and CD8^+^ T cells with a CD44^low^ phenotype from normal mice. Based on the real-time RT-PCR, a significant induction of IFN-γ was detected in DP cells from *T. cruzi* infected mice, at comparable levels of CD44^high^ activated CD4^+^ and CD8^+^ T cells. As expected, naïve CD44^low^ single-positive CD4^+^ and CD8^+^ T cells obtained from uninfected control mice did not exhibit IFN-γ mRNA induction (Fig. 6b).

To further study the differentiation status of DP cells, we tested whether this particular T cell subset had cytotoxic activity. To address this issue, DP cells were purified by cell sorting from chagasic peripheral lymph nodes at day 15 post-infection and varying numbers of sorted DP cells were incubated with P815 target cells. The effector:target (E:T) ratio was normalized based upon the frequency of T cells in order to compare the cytolytic activity on a per cell basis. Anti-CD3 mAb-redirected cytotoxic activity was measured because specific antigens for DP cells are unknown at present. In contrast to naïve CD4^+^ and CD8^+^ T cells, DP cells showed significant cytotoxic activity (Fig. 6c), which was specific for the anti-CD3 mAb signal, since P815 cell incubated in the absence of the antibody did not induce any cytotoxic activity (data not shown).

We further compared the cytotoxic activity of DP cells and activated CD8^+^ T cells obtained from peripheral lymphoid tissues at day 15 after *T. cruzi* infection. In these experiments, activated CD8^+^ T cells from chagasic peripheral lymph nodes were sorted by FACS based on the expression of high levels of the CD44 activation marker. As control, we purified naive CD8^+^ T cells with a CD44^low^ phenotype from normal mice. Our data show that the level of cytotoxic activity from DP T cells were reduced as compared to activated CD44^high^CD8 T cells but significantly higher than naïve CD44^low^CD8 T cells (Fig. 6d).

**Figure 7:**
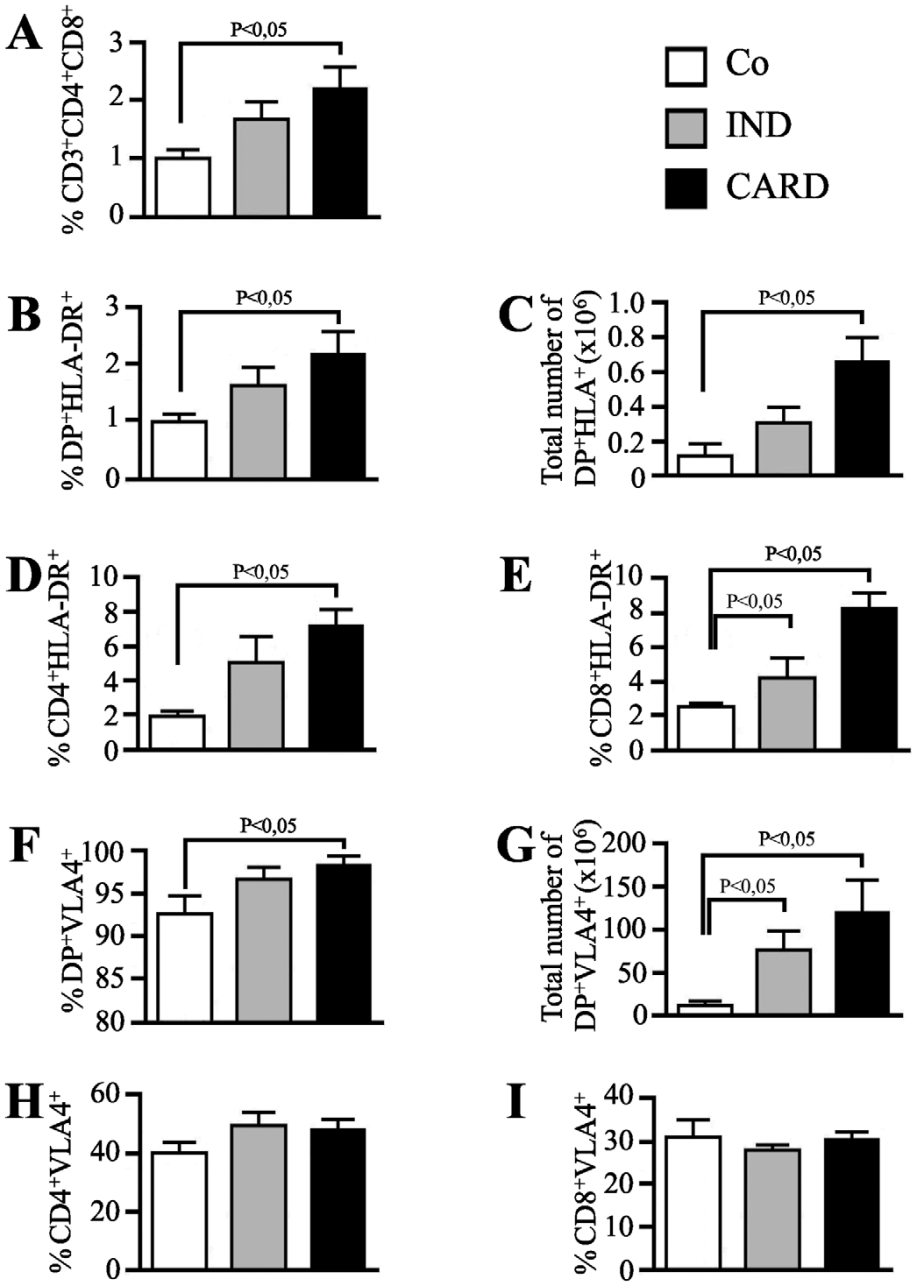
Frequency and phenotype of peripheral blood CD4+CD8+ T cell subset from chronic chagasic patients. Peripheral whole blood from non-infected individuals (NI), patients at the indeterminate (IND) and cardiac (CARD) forms of Chagas disease were analyzed by four-color flow cytometry for expression of CD3, CD4, CD8, HLA-DR and VLA-4 markers (n = 15 individuals per group). **(a)** The percentages of CD4+CD8+ cells on CD3+ T lymphocytes are indicated for each histogram. The frequency **(b)** and total number **(c)** of activated DP T cells, frequencies of **(d)** single-positive CD4 T cells and **(e)** CD8 T cells are shown by assessing the expression of HLA-DR. The frequency **(f)** and total number **(g)** of activated DP T cells, frequencies of **(h)** single-positive CD4 T cells and **(i)** CD8 T cells are shown by assessing the expression of VLA-4. The differences between the groups were considered significant at *p* less than 0.05 and are represented by p<0.05.

In the light of evidence that the DP cells released into the periphery acquire an activated phenotype similar to what is described for activated effector or memory single-positive T cells, we next evaluated a possible relationship between the presence of these cells and the development of severe clinical forms of human Chagas disease. For this purpose, we first examined the frequency of peripheral blood DP cell subset in cross-sectional studies of chronic chagasic patients at the indeterminate or cardiac clinical forms of Chagas disease. The results show higher percentage of DP cells from peripheral blood of cardiac chagasic patients as compared to noninfected individuals. However, this statistically significant increase was not seen in the percentages of DP cells from peripheral blood of patients at the indeterminate stage of chonic disease, when compared to control individuals (Fig. 7a).

Moreover, we showed a statistically significant increase in the activation profile of extrathymic activated DP cells in cardiac chagasic patients to assess the expression of activation marker HLA-DR indicated that peripheral blood subset of DP cells obtained from chronic cardiac chagasic patients (but not from patients at the indeterminate form of the disease) highly express significant levels of this molecule, as compared to normal individuals (Fig. 7b). Furthermore, the increase of extrathymic HLA-DR^+^ DP cell population in patients having the cardiac form of human Chagas disease was also correlated with the augmentation of the frequencies of activated HLA-DR^+^ single-positive CD4^+^ T cell (Fig. 7c) and CD8^+^ T cell subsets (Fig. 7d) obtained from chronically infected chagasic patients. CD8^+^ T cells also exhibited an activated phenotype regarding to the HLA-DR^+^ expression in the peripheral blood of patients at the indeterminate form of the disease (Fig. 7d).

Additionally, phenotype FACS analysis of the expression marker VLA-4 indicated that the peripheral blood subset of DP cells from both indeterminate and cardiac chronic patients express highed levels of this molecule as compared to normal individuals (Fig. 7e). Interestingly, we showed that there is no modulation on the expression of this marker in single-positive CD4^+^ cell (Fig. 7f) and CD8^+^ T cell (Fig. 7g) subsets obtained from chronic cardiac patients. Taken together, these results indicate that increased percentages of circulating DP cells are associated with severe clinical form of chronic human Chagas disease. While the percentages of these cells are not significantly increased in chronic patients at the indeterminate form, as the disease progresses to a cardiac clinical form, the peripheral blood subset of DP cells acquire a full activated HLA-DR^high^VLA-4^high^ profile.

## Discussion

Several pathogens, including *T. cruzi*, cause thymic atrophy [30]. Although the precise mechanisms underlying this phenomenon are not completely elucidated, it is most likely that this event is linked to a particular pathogen-host relation established during infection. Based on the studies developed with the *T. cruzi* model, it has been shown that the inflammatory syndrome mediated by TNF-α during the acute phase of infection induces the activation of hypothalamus-pituitary-adrenal (HPA) axis with the consequent release of corticosterone. In its turn, the glucocorticoid rise is likely associated with profound effects on the changes observed in the thymuses of *T. cruzi* infected mice, including the lymphoid and non-lymphoid compartments, as well as upon the disease outcome [31], [32]. Nevertheless, it should be pointed out that other host-derived molecules can be involved in the *T. cruzi*-induced thymocyte depletion. This is the case for galectin-3: thymic atrophy is not seen in *T. cruzi*-infected galectin-3 knockout mice [33]. Moreover, the parasite-derived trans-sialidase is involved in the generation of intrathymic T cell death [5], [34]. In a second vein, the thymic microenvironmental changes seen after acute infection comprise the enhanced expression of extracellular matrix ligands and receptors, which correlates with a higher fibronectin-driven migration of DP thymocytes, and the abnormal raise of immature DP cells in lymph nodes [8], [9].

Nevertheless, it was not determined if the changes of the thymic microenvironment seen following *T. cruzi* infection, would also lead to an altered intrathymic negative selection of the T cell repertoire. There is an emerging consensus stating that clonal deletion normally occurs late in thymocyte development at the double-positive to single-positive transition, and is believed to take place at the cortico-medullary junction of the thymic lobules [35]. In this regard, it has been shown that the thymic metallophilic macrophages restrictedly located at the cortico-medullary junction are related to the process of thymocyte maturation [23]. These cells show a number of features that differentiate them from both cortical macrophages and medullary interdigitating cells. They possess extremely developed specialized endocytic compartments for the processing and presentation of antigens by MHC class II molecules [24]. Here, we demonstrate that, although the alterations observed in the cortex and medullary compartment reveal the severe atrophy of the organ during the acute infection, the changes promoted by the infection in the thymic architecture changed the topological distribution of the metallophilic macrophages. Indeed, these cells are also found in the cortico-medullary region but in higher numbers and are spread along the cortex, as compared to controls. The distribution of these cells in the thymic compartments may represent a functional shift for the clonal deletion from the cortico-medullary junction to the cortex during the acute phase of the infection.

It is largely established that interactions between TEC and thymocytes control the development of the thymic microenvironment and T cell development. Previous studies have shown that the disruption of normal thymic architecture is known to affect the expression pattern of autoantigens by TEC and functionality of thymus [36]-[38]. Thymic medullary atrophy and decreased expression of Aire and TRAs have been reported in mouse models deficient in several genes involved in the NFκB pathway, such as TRAF6, NIK, RelB or p52, suggesting an important role of this pathway in the development of thymic medulla [12], [39]. We showed that the expression of Aire and highly selective tissue restricted antigens was readily detectable in whole thymus by real-time PCR analysis from infected mice, rather similar to controls. These data suggest that the expression of peripheral antigens in the infected thymuses is sufficient to modulate the tolerance induction by the negative selection process.

As the acute phase of infection progresses, the thymic atrophy becomes evident, as is the increase in numbers of apoptotic intrathymic DP cells, compared to their respective normal counterparts [4], [5]. Although this phenomenon may be a consequence of the changes observed in the organ, our data show that along the DP depletion there is sustained expression of Bim, an pro-apoptotic factor essential for thymocyte negative selection. Finally, by using an OTII TCR transgenic system, we were able to demonstrate that the administration of the cognate OVA peptide in the acutely infected mice undergoing thymic atrophy can induce TCR-stimulation-induced apoptosis of semi-mature thymocytes. Collectively, these data point out that negative selection operates normally during infection-promoted thymic atrophy, since the DP cells can be negatively-selected in the infected thymus by antigen-induced depletion. This supports previous work showing that intrathymic mature single-positive CD4^+^ or CD8^+^ T cells do not bear forbidden TCR genes as compared with their DP counterparts undergoing intrathymic differentiation [10].

Although the intrathymic checkpoints necessary to avoid the maturation of T cells expressing a forbidden T cell receptor repertoire are present in the acute phase of murine Chagas disease, it has been shown that DP cells are early released from infected thymus to the periphery [7], [9]. Exit of DP cells from thymus during both acute and chronic *T. cruzi* infection [10], may be favored by the upregulation of CD62L (L-selectin), which controls lymphocyte homing to lymph nodes. Since DP cells are progressively accumulated in peripheral lymphoid organs of *T. cruzi* acutely infected animals, we determined whether those cells exhibit an activated profile similar to effector/memory single positive T cells. The existence of this unconventional and rare (<5%) lymphocyte population in the periphery was explained as a premature release of DP cells from the thymus into the periphery, where their maturation into functionally competent single-positive cells continues [10]. There is, however, considerable evidence of an increased frequency of peripheral CD4^+^CD8^+^ T cells during viral infections and during acute *T. cruzi* infection. For example, in human immunodeficiency virus or Epstein-Barr virus infections, the percentage of DP cells can increase to 20% of all circulating lymphocytes [40], [41]. This fluctuation is also present in the secondary lymph nodes as we demonstrated in the experimental model of Chagas disease, in which DP cell subset increases up to 16 times in subcutaneous lymph nodes [10].

Interestingly, despite the expansion of peripheral DP lymphocytes in the experimental model of Chagas disease, these cells develop an activated phenotype, upregulating the activation markers CD44 and CD69, tightly linked to the differentiation status of T cells. In addition, we showed that highly purified (>98%) DP cell populations from infected mice obtained after cell sorting, produced high levels of IFN-γ mRNA. Furthermore, similar to previous studies showing high cytotoxic activity and effector memory phenotype of extrathymic DP cells in *cynomolgus* monkeys [42] and in a chimpanzee with experimental hepatitis C virus infection [43], our results indicate that the DP cells purified from peripheral lymphoid tissues of chagasic animals show cytotoxic activity as compared to naïve single-positive CD4^+^ or CD8^+^ T cells. Most interestingly, we found that patients having the cardiac form of human Chagas disease presented higher percentages of peripheral blood HLA-DR^+^ DP T cells as compared to non-infected individuals. These data suggest that this T cell subset might be associated with the development of the cardiac clinical form of the disease, probably as activated cells.

All together, our results indicate a role of the DP T cell subset in the inflammatory process caused by parasite-driven immune responses. It is possible that co-expression of CD4 and CD8 molecules on the T cell membrane would enhance the avidity of the TCR to its target cell. In this way, the concomitant trigger of CD4 and CD8 co-receptors through MHC-antigen complex would reduce the threshold of the antigen signal and decrease the requirements for the costimulatory signals to generate T cell activation [44]. This cascade of events would favor the DP T cell activation in the presence of low antigen concentrations. This scenario could promote a quick activation of the peripheral DP subsets upon the onset of infection where the antigens derived from the parasite are limiting. It is thus plausible to conceive that these cells may play a relevant role in modulating the adaptive immune responses via cytokine secretion. Accordingly, the cytokine profile secreted by DP T cells may drive the function of dendritic cells during the adaptive immune responses, thereby providing a link between innate and adaptive immunity. In addition, DP cells may have regulatory roles in host immunity. As DP cells are theoretically recognizing both class I and class II MHC complexes, it is also possible that these cells may eliminate activated antigen presenting cells, which would favor the parasite during of infection.

In conclusion, our data indicate that the key intrathymic checkpoints necessary to promote negative selection process of thymocytes are effective during acute chagasic thymic atrophy. Although the negative selection process is still functional in the acute phase, DP cells are prematurely released to the periphery. During the course of experimental infection, these peripheral DP cells acquire an activated phenotype similar to what is described for activated and memory single-positive T cells with high IFN-γ production, CD44^+^CD69^+^ expression and cytotoxic activity. This is particularly important, as we show for the first time that the appearance of full activated peripheral DP cells correlate with the cardiac clinical form of human chronic Chagas disease. Most likely, the presence of peripheral, mature and activated DP lymphocytes challenges the perception of the T cell populations involved in adaptive immune responses during the *T. cruzi* infection and suggests that DP cells promptly participate in the immune response in *T. cruzi* infection. Therefore, although the function of this DP T cell population remains to be defined *in vivo*, the presence of peripheral activated DP cells with potentially autoreactive TCR may contribute to the imunopathological events found in both murine and human Chagas disease. Correlations between the changes in the levels of DP T cell subsets and the extent of myocardial lesion during the evolution of cardiac disease could identify a clinical marker of disease progression and may help in the design of alternative therapies for the control of chronic morbidity of chagasic patients.
